# Editorial to “Long‐term outcomes of ventricular tachycardia ablation in repaired tetralogy of Fallot: systematic review and meta‐analysis”

**DOI:** 10.1002/joa3.13127

**Published:** 2024-08-05

**Authors:** Taisuke Nabeshima, Naokata Sumitomo

**Affiliations:** ^1^ Department of Pediatric Cardiology Saitama Medical University International Medical Center Hidaka Saitama Japan

Today, most repaired tetralogy of Fallot (rTOF) patients survive to adulthood because of advances in medical and surgical treatments. As survival rates improve, however, long‐term complications such as VT remain significant challenges. A report described ventricular arrhythmia occurring in 44% of postrepair patients, noting that a higher age at repair correlated with an increased incidence of life‐threatening events, mostly because of sustained VT. Risk stratification and effective methods of catheter ablation (CA) for VT to prevent further recurrence are the major clinical demands to which Jagannatha et al. tried to answer in this report.^1^

In their work, first, Jagannatha et al. analyzed risk factors for VT in rTOF patients.^1^ According to the AHA guideline, risk factors for SCD include LV systolic or diastolic dysfunction, nonsustained VT, QRS duration >180 ms, extensive RV scarring, and inducible sustained VT at electrophysiologic study (EPS).^2^ Primary prevention ICD therapy is recommended for rTOF patients with multiple risk factors for SCD (class IIa), but CA for these patients is not mentioned.^2^ According to the JCS guideline, CA is recommended only for patients weighing more than 15 kg when medication therapy has failed (class IIa).^3^ The recommendation level for CA is not high because its efficacy has not been proven in this population so far.

On the contrary, favorable opinions of CA for patients with ischemic heart disease are growing nowadays. Comparison of CA versus antiarrhythmic drugs (AAD) after ICD therapy for ischemic cardiomyopathy is being addressed in two trials: the ongoing VANISH2 and the recently published SURVIVE‐VT trial. In the latter, substrate‐based CA reduced the composite endpoint of cardiovascular death, appropriate ICD shock, hospitalization because of heart failure, or severe treatment‐related complications compared with AAD therapy. These favorable outcomes are mainly because of the technological advancements in the electroanatomical mapping system (3D‐EAM) and our deepened understanding of the relationship between the myocardial substrate and the mechanism of clinical VT. Among several findings indicating the location of critical isthmus based on substrate mapping, a slow‐conducting anatomical isthmus (SC‐AI) is one of the most promising indicators. Kapel et al. established a threshold of <0.5 m/s for the identification of most induced VT circuits, achieving 93% sensitivity and 100% specificity, thereby becoming the gold standard for SC‐AI identification.^4^

In the study by Jagannatha et al., the presence of SC‐AI was added to the risk factors for SCD in rTOF patients. More importantly, the study demonstrated the efficacy of VT ablation in rTOF patients, particularly when based on SC‐AI. According to their report, the prevalence of VT in this population is 21.4%, and among the 397 patients who underwent 3D‐EAM, 121 (30.4%) had SC‐AI. Of note, not all the SC‐AIs were related to VT inducibility, with 3.7% showing no correlation. In a mean follow‐up of 40 ± 21 months, VT recurrence after SC‐AI‐based CA occurred in only 2 out of 70 cases, demonstrating a 10‐fold decrease in recurrence compared with conventional methods (RR: 0.11; 95% CI: 0.03–0.33).^1^

Recently, the concept of SC‐AI‐based VT ablation has been applied to automated isochronal late activation mapping (ILAM). Using a multielectrode catheter and specific software that annotates the offset of the latest component at each local electrogram, automated ILAM visualizes eight equally distributed activation isochrones. Deceleration zones (DZs) are defined as regions with more than three isochrones within a 1 cm radius, correlating with the region of conducting slowing below the normal limit (<0.6 m/s). E. Arana‐Rueda et al. reported that ILAMs also allow accurate identification of SC‐AI in patients with rTOF, achieving 90% sensitivity and 100% specificity.^5^ In this study, ILAM was performed on 14 rTOF patients during sinus rhythm. Of these, 10 cases were positive for DZs, and nine had SC‐AIs. VTs were induced only in SC‐AI‐positive patients (seven cases, 77%), and DZs co‐localized with the critical isthmus of induced VTs in 88% of cases. Eighty percent of SC‐AIs were located within anatomical isthmus^3^ (between the pulmonary valve and VSD patch), identifying this area as the main source of the slow conducting zone as previously reported.^5^

From these points of view, we might also speculatively address the role of SC‐AI‐based ablation prior to procedures like transcatheter pulmonary valve implantation (TPVI). Given the anatomical changes post‐TPVI, areas covered by the device may become inaccessible, making prophylactic ablation a strategic consideration to prevent VT in vulnerable sites.

However, despite these technological advances and encouraging outcomes, several challenges remain. The sensitivity of detecting clinical VTs using SC‐AIs needs enhancement. Often, multiple pacing sites are required to reveal hidden SC‐AIs, and adjunctive imaging techniques like late gadolinium enhancement (LGE) in cardiac MRI may be necessary to detect deeper myocardial SC‐AIs.

Furthermore, there is a pressing need to clarify when and how the myocardium, especially within anatomical isthmus,^3^ might degenerate into SC‐AIs over time because of conditions like moderate‐to‐severe pulmonary regurgitation. This potential for degeneration could be linked to an overall increase in myocardial fibrosis, as indicated by LGE in CMR, which itself is a known risk factor for SCD.

In conclusion, while the landscape of VT management in rTOF patients is rapidly evolving, it is crucial to establish a more robust evidence base for SC‐AI‐based ablation in rTOF patients. This entails not only refining the existing technologies and techniques but also conducting long‐term follow‐up studies to assess the durability of these interventions.

## FUNDING INFORMATION

None.

## CONFLICT OF INTEREST STATEMENT

None.

## ETHICS STATEMENT

None.

## PATIENT CONSENT STATEMENT

None.

## CLINICAL TRIAL REGISTRATION

None.

## Data Availability

Nothing to declare.
